# Modelling the Mechanical and Strain Recovery Behaviour of Partially Crystalline PLA

**DOI:** 10.3390/polym11081342

**Published:** 2019-08-13

**Authors:** John Sweeney, Paul Spencer, Karthik Nair, Phil Coates

**Affiliations:** IRC in Polymer Science and Technology, Mechanical and Energy Systems Engineering, Faculty of Engineering and Informatics, University of Bradford, Bradford BD7 1DP, UK

**Keywords:** PLA, strain recovery, modelling, finite element method, crystallinity

## Abstract

This is a study of the modelling and prediction of strain recovery in a polylactide. Strain recovery near the glass transition temperature is the underlying mechanism for the shape memory in an amorphous polymer. The investigation is aimed at modelling such shape memory behaviour. A PLA-based copolymer is subjected to stress–strain, stress relaxation and strain recovery experiments at large strain at 60 °C just below its glass transition temperature. The material is 13% crystalline. Using published data on the mechanical properties of the crystals, finite element modelling was used to determine the effect of the crystal phase on the overall mechanical behaviour of the material, which was found to be significant. The finite element models were also used to relate the stress–strain results to the yield stress of the amorphous phase. This yield stress was found to possess strain rate dependence consistent with an Eyring process. Stress relaxation experiments were also interpreted in terms of the Eyring process, and a two-process Eyring-based model was defined that was capable of modelling strain recovery behaviour. This was essentially a model of the amorphous phase. It was shown to be capable of useful predictions of strain recovery.

## 1. Introduction

Polylactic acid (PLA) and related polymers and blends are a focus of increasing attention, arising both from their potential for degradability within the natural carbon cycle for disposable products [1,2], and from their bioresorbable properties for prosthetic implants [3,4,5,6]. In the latter category, there are applications for ‘smart’ implants that make use of the polymer’s shape memory properties [7]. As a result, of the load bearing nature of many applications, both potential and realised, it has become important to understand the mechanical behaviour of PLA. This is the motivation of this paper, for which a fundamental study was made of the mechanical behaviour of PLA, with particular emphasis on aspects relevant to shape memory and strain recovery.

The physical and mechanical properties of PLA materials have recently been reviewed by Farah et al. [8] and Garlotta [9], the latter with more emphasis on crystalline properties. Bergström and Hayman [10] have produced a review concentrating more on mechanical properties with relevance to medical applications. PLA materials resemble most other thermoplastics in that they are mechanically nonlinear, time-dependent and have varying levels of crystallinity. At elevated temperatures near the glass transition of around 60 °C, they are capable of being deformed to large strains, acquiring molecular orientation and hence shape memory properties. There have been two notable recent studies of molecular orientation effects arising from biaxial deformations [11,12].

PLA materials are thermoplastics that can be processed using well-established conventional methods including extrusion, injection moulding, injection stretch blow moulding, film or sheet casting and thermoforming [2]. They have also been made into foams to produce superhydrophobic devices for oil–water separation [13]. Additionally, they are a leading polymer in additive manufacturing [14]. To induce shape memory properties into the material, a manufacturing process that creates high levels of molecular orientation is required. Such a method is die drawing, originally developed to produce polymer products with high stiffness and high strength properties [15,16,17]. This process has been successfully applied to PLA-based polymers [18,19] providing a process with potential for shape memory polymer production.

In addition to applications of the pure polymer, PLA has also been used as the principal component of biocompatible blends and composites. Vasile et al. [20] have plasticized PLA with polyethylene glycol (PEG) and used melt-mixing to combine it with biological materials including chitosan in various combinations. The resulting materials showed useful levels of antioxidant and antibacterial activity together with good biocompatibility, with potential for both biomedical and packaging applications. PLA/PEG systems also have a role in the production of polymer-nanoparticles hydrogels [21]. Well-dispersed graphene nanocomposites have been produced using poly(butylene adipate-co-terephthalate) (PBAT) using a PEO/PLA/PBT system [22].

This study is motivated by growing developments in biocompatible shape memory polymers in biomedical applications [23]. Recent examples of specific applications of shape memory PLA systems range from in-vivo devices made from shape memory nanocomposites [24] to melt-blown nonwoven fabrics with potential for load bearing implants, scaffolds and wound dressings [25]. The level of crystallinity is in general an important factor associated with high stiffness and strength, and nucleation techniques have been developed to enhance it in PLA systems [26]. However, in the biomedical context, it has been established that the bioresorbability of poly l-lactide (PLLA) is too low due to its high crystallinity [27], and so less crystalline forms are used. Such a material, a polylactide with a relatively small (~13%) level of crystallinity, is the subject of the experimental study in this paper. The objective is to develop a material model that predicts strain recovery after tensile extension, to form the basis of a model of the shape memory effect. Shape memory is driven by the entropic stresses in the amorphous phase of the polymer. To understand this mechanism on the basis of mechanical experiments on the partially crystalline polymer, it is necessary to assess the effects of the crystalline phase on the measurements. This we do by modelling in a way that shows a significant effect of the crystal phase on the macroscopic elastic properties. Extension of the modelling and comparison with stress–strain experiments allow us to isolate the yield behaviour of the amorphous material and define its strain rate dependence. A constitutive model is then constructed that enables the stress relaxation and recovery behaviour to be simulated and compared with experimental observations of strain recovery. The experiments are a direct measure of shape memory effectiveness and thus an appropriate means of evaluating the model. A testing temperature of 60 °C is chosen as, being slightly below the glass transition temperature, it makes possible high levels of molecular orientation and also enables subsequent strain recovery, replicating shape memory behavior.

The material modelling to assess the effects of the crystalline phase is based on the concept of crystallites as inclusions within an amorphous matrix. This approach to the modelling of semicrystalline polymer was pioneered by Halpin and Kardos [28], who made use of the Halpin–Tsai equations [29]. Halpin and Kardos used an approach that acknowledged the effect of the stiffness of crystals relative to that of the amorphous phase. This gave better predictions than previous models, which had treated crystals as merely sources of more crosslinking sites. Working with crystal volume fractions of up to 30%, they used the Halpin–Tsai composite theory to produce successful models of the stiffness of polyolefins. There is a potential objection to the modelling of crystallites as inclusions isolated from each other, which Halpin and Kardos discussed; the existence of tie-molecules. Partly as a result of the success of their modelling, they describe the contribution of tie-molecules as “at best, of only secondary importance” [28]. It is clear that, at small material strains, tie-molecules will behave no differently than other molecules in the amorphous phase. They will only have an effect of linking crystals to form a network when they are highly extended at large strains, and we are using this form of modelling only up to a strain of 0.025. As a consequence of these arguments, we shall proceed with modelling the material as isolated crystallites within an amorphous matrix.

## 2. Materials and Methods

The material is a copolymer of l-lactide and dl-Lactide in a 70:30 molar ratio. It was supplied in the form of granules and processed using melt extrusion into hollow tube to be used as specimens for mechanical testing.

Before processing the polymer, granules were dried overnight in a vacuum oven. A single-screw Dr Collin extruder (Maienbeth, Germany) with a 16 mm diameter screw and the final heating zone at 180 °C was used to extrude the tube material. Granules were supplied into the hopper, in which nitrogen was circulated to minimise moisture. The extrudate was pulled through a cooling bath kept at 20 °C by means of a caterpillar. The tube was 3.2 mm in external diameter and 0.8 mm internal diameter.

DSC testing was performed on the extruded material using a TA Instruments (New Castle, DE, USA) Discovery DSC Instrument operating at a heating rate of 10 °C/min. From these results the crystallinity was calculated as 13.1% using the method of Kong and Hay [30], and the glass transition temperature estimated as 64.3 °C. The melt temperature was 165 °C.

Molecular mass of the tube material was determined using gel permeation chromotography (Waters Acquity Advanced Polymer Chromatography System, Milford, MA, USA). The values were measured as ${\bar{M}}_{w}=650,000$ Da and ${\bar{M}}_{n}=370,000$ Da.

Mechanical tests were carried out in a fan oven at 60 °C using an Instron 5565 testing machine (High Wycombe, UK). Tensile test specimens consisted of lengths of tube with gauge length 75 mm. Specimens were stretched at a range of constant speeds to a strain of 1 (extension ratio 2). For all experiments, tensile stretching was performed at 60 °C at constant speed with strain rates in the range of 2.2 × 10^−3^–4.0 × 10^−2^ s^−1^. Tensile tests were of two kinds: stress relaxation, for which the extension was held constant and the load monitored for a set time interval; and strain recovery, for which one end of the specimen was released from the testing machine on attaining the set extension and strain then allowed to recover under fixed load. For the latter tests, the specimen was released by means of a spring-loaded pin and strain recovery was monitored using video extensometry through the oven window. Images were captured using a PixeLINK model PL-D722MU-T video camera (Rochester, NY, USA), and images were analysed using the Fiji image processing software package based on ImageJ (Eliceiri, University of Wisconsin-Madison, Madison, WI, USA) [31].

Three-point bend tests were carried out on lengths of tube specimen loaded centrally on a span of 25 mm using the same testing machine, oven and temperature using. Testing speeds were such as to provide strain rates close to those in the tensile tests.

## 3. Results

### 3.1. Small Strain Structural Modelling

#### 3.1.1. Analytical Approach

The Mori-Tanaka model [32,33], based on the exact solution for an ellipsoidal inclusion within a continuous medium, is a more rigorous approach to composite modelling than the semi-empirical Halpin–Tsai method. The Mori–Tanaka composite is assumed to consist of regularly spaced ellipsoidal inclusions. Each ellipsoid is a spheroid with two equal axes, axisymmetric at the third axis (the symmetry axis). The inclusions are arranged with the symmetry axes aligned parallel to one another. The aspect ratio *a* is the ratio of the length of the symmetry axis to that of the other two axes; thus, both disc-like (*a* < 1) and rod-like (*a* > 1) geometries can be defined. Both matrix and inclusions are of isotropic elastic material. We have adopted the approach to the Mori–Tanaka theory established for ellipsoidal inclusions [34,35].

The amorphous and crystal phases are assigned elastic moduli *E*_a_ and *E*_c_ respectively, with corresponding Poisson’s ratios ν_a_ and ν_c_. As a result of the alignment of the ellipsoids, the composite as a whole is orthotropic, with modulus along the direction of the ellipsoids’ symmetry axes $E_{∥}$ given by

(1) $$\frac{E_{∥}}{E_{a}}=\frac{A}{A+\phi \left(A_{1}+2\nu_{a}A_{2}\right)}$$

and the modulus normal to it by

(2) $$\frac{E_{⊥}}{E_{a}}=\frac{2A}{2A+\phi \left(-2\nu_{a}A_{3}+\left(1-\nu_{a}\right)A_{4}+\left(1+\nu_{a}\right)A_{3}A\right)}$$

Here ϕ is the volume fraction of inclusions and *A*, *A*_1_, *A*_2_, *A*_3_, and *A*_4_ are functions of Eschelby’s tensor, the elastic constants of both phases, and ϕ [34,35].

**Figure 1:**
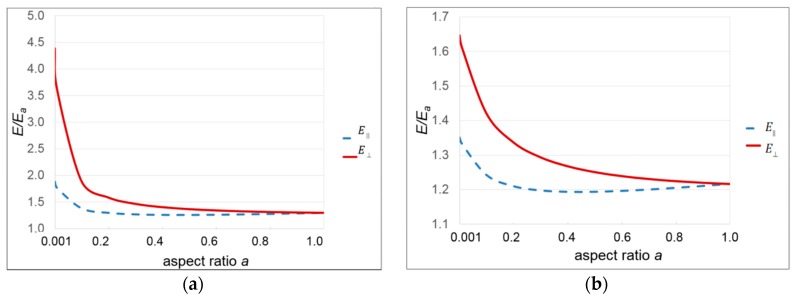
Mori–Tanaka Modulus predictions for (**a**) *E*_crystal_ = 14 GPa and (**b**) *E*_crystal_ = 3 GPa.

As discussed further in Section 3.3.1 below, normal moduli of the α-phase PLLA crystals have been measured using X-ray techniques [36]. Strains in the crystals are deduced from the change in lattice spacing as measured by X-ray diffraction when the material is subject to a known stress. For the *a*, *b* and *c* axes of the orthorhombic crystal, the Young’s moduli are respectively 3.2, 2.8 and 14 GPa, where the chain axis is along *c*. In a lamellar crystal structure, the chain axis is normal to the plane. In terms of the Mori–Tanaka model, such a structure would be best approximated by disc-like ellipsoids, with the symmetry axis of each ellipsoid identified with the chain axis, which also corresponds to the direction of the largest modulus. However, the theory is limited to isotropic ellipsoids and so we shall explore the extremes *E*_crystal_ = 3 and *E*_crystal_ = 14 GPa modulus in isotropic ellipsoids. In Figure 1, we present predictions of moduli in terms of the ratios of Equations (1) and (2) for disc-like inclusions over a range of aspect ratios *a* < 1. Here we assume that the amorphous modulus *E*_a_ = 0.5 GPa, consistent with the modulus of the bulk material in 3.2.1 below. We take the volume ratio to be ϕ = 0.13 as measured for our material by DSC, and Poisson’s ratios of 0.4 for both materials.

It is clear that the crystals can have a significant effect on the modulus at this concentration. This is consistent with the dynamic mechanical measurements of Tábi et al. [37]. However, in this model the alignment of the ellipsoids ensures that the modulus increase is accompanied by an unrealistic level of anisotropy as defined by $E_{∥}$ and $E_{⊥}$. The choice of modulus also has significant effects. This suggests that the issue should be pursued further using a model in which the inclusions are elastically anisotropic and randomly aligned.

#### 3.1.2. Numerical Approach

**Figure 2:**
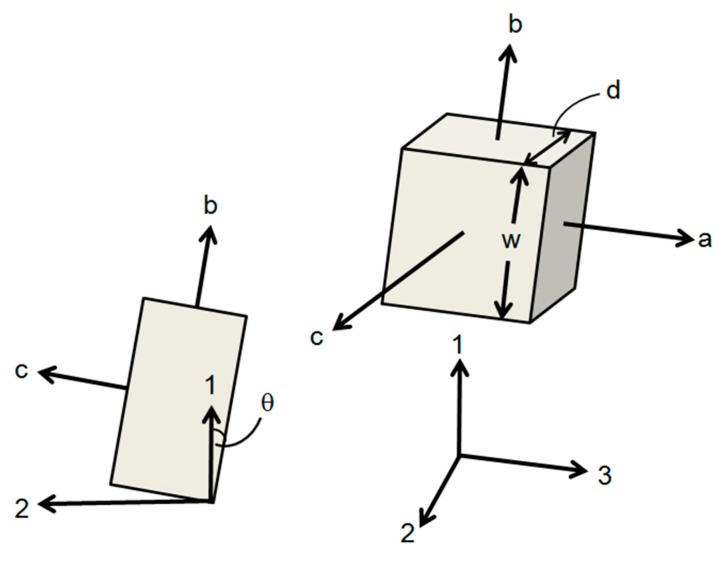
Orientation of model crystallite with respect to global 1, 2, 3 axes coincident with the RVE cube axes. The a crystallite axes are all parallel to the 3 axis and the each crystallite rotates about the a axis in the 1–2 plane to the angle θ. The c axis is the (stiff) chain axis. The crystallite is assumed to be transversely isotropic in the ab plane. Left: view along the 3 axes. Right: general view.

For a more realistic method, we have created three-dimensional finite element models of the partially crystalline structure. Solid orthorhombic regions representing the crystallites are embedded within a cube of material representing the amorphous polymer, to give a representative volume element (RVE). Model crystallites of identical size and shape are added to the structure at random positions and at random orientations about one of the global axes, and are ensured to be non-intersecting using an acceptance–rejection algorithm [38]. Thus, the orientation of each crystallite is semi-random, in that one set of edges is parallel to the 3 axes but otherwise the shape is angled randomly in the 1–2 plane; see Figure 2. Orthotropic elastic properties based on X-ray measurements on PLA crystals [36] are assigned to the model crystallites. To simulate an infinite body and avoid unrealistic deformations at the RVE boundaries, periodic displacement boundary conditions are applied at the cube surfaces. The overall stress response is evaluated using the ABAQUS finite element package (ABAQUS 6.14-2, Dassault Systèmes, Johnston, RI, USA). Small-strain elastic properties of the composite solid are derived using the stress responses to appropriate boundary conditions applied to the models averaged over repeated realisations. Uniaxial yield properties are also explored at larger strains using the same RVEs with elastic–plastic matrix properties. This enables the overall yield response of the composite solid to be quantitatively related to the yield point of the matrix.

Periodic boundary conditions are enabled by designing the RVE mesh so that there are matching node positions on opposite boundary faces. For an RVE of side length L, for boundary faces normal to the 1 direction $X_{1}=0$ and $X_{1}=L$ the nodal positions are such that for every node at $\left(0,X_{2},X_{3}\right)$ there is a corresponding node at $\left(L,X_{2},X_{3}\right)$ and vice versa. Similar conditions apply for the pairs of faces $X_{2}=0,\;X_{2}=L$ and $X_{3}=0,\;X_{3}=L$.

To evaluate elastic properties, normal moduli are calculated by deforming the RVE along each global direction in turn while restraining boundary planes parallel to this direction of deformation. For an extension ΔL along 1, the displacements $u_{1}$ at the boundary planes normal to 1 are related by

(3) $$u_{1}\left(L,X_{2},X_{3}\right)-u_{1}\left(0,X_{2},X_{3}\right)=\Delta L$$

for each pair of corresponding nodes. Plane strain type restraints in the 2 and 3 directions are defined by

(4) $$u_{2}\left(X_{1},L,X_{3}\right)-u_{2}\left(X_{1},0,X_{3}\right)=u_{3}\left(X_{1},X_{2},L\right)-u_{3}\left(X_{1},X_{2},0\right)=0$$

The systems of Equations of (3) and (4) are programmed into ABAQUS. Analogous systems are created for plane strain deformations of the same magnitude along 2 and 3. Each randomly generated realisation of the model is subject to the same three deformations along 1, 2 and 3. The macroscopic stress responses $\sigma_{1},\;\sigma_{2}$ and $\sigma_{3}$ calculated in the analysis then define a stiffness matrix **C** for each realisation of the RVE. For the macroscopic strain given by ε=ΔL/L, Hooke’s Law gives for the 1, 2 and 3 directions, respectively,

(5) $$\begin{array}{l} \sigma_{1}=c_{11}\varepsilon ,\;\sigma_{2}=c_{12}\varepsilon ,\;\sigma_{3}=c_{13}\varepsilon \\ \sigma_{1}=c_{12}\varepsilon ,\;\sigma_{2}=c_{22}\varepsilon ,\;\sigma_{3}=c_{23}\varepsilon \\ \sigma_{1}=c_{13}\varepsilon ,\;\sigma_{2}=c_{23}\varepsilon ,\;\sigma_{3}=c_{33}\varepsilon \end{array}$$

thus defining the components *c*_ij_ of **C**. The stiffness matrix **C** is inverted to give a compliance matrix **S**, the diagonal terms of which give the moduli $E_{1},\;E_{2}$ and $E_{3}$ along global directions 1, 2 and 3, respectively.

For a given set of conditions, several RVEs are generated and solved to give a set of modulus values, allowing a mean and standard deviations to be obtained. A specified crystal volume fraction φ corresponds to a total number *N* of inclusions. To represent the stochastic nature of the material, the number of inclusions *n* in each realisation is derived from the expected value *N* on the basis of a Poisson distribution, such that the probability of *n* inclusions is given by

(6) $$p_{n}=\frac{N^{n}}{n!}e^{-N}$$

where the average of *n* tends to *N* over a large number of realisations.

The yield properties of the composite system have been explored using uniaxial deformations of selected RVEs. Here the inclusions retain their elastic properties, but the matrix is assumed to be elastic-plastic with a simple von Mises yield criterion. This takes the form

(7) $$2\sigma_{Y}={\left({\left(\sigma_{I}-\sigma_{\mathrm{II}}\right)}^{2}+{\left(\sigma_{\mathrm{II}}-\sigma_{\mathrm{III}}\right)}^{2}+{\left(\sigma_{\mathrm{III}}-\sigma_{I}\right)}^{2}\right)}^{1/2}$$

for uniaxial yield stress $\sigma_{Y}$ and principal stresses $\sigma_{I},\;\sigma_{\mathrm{II}}$ and $\sigma_{\mathrm{III}}$. This enables us to explore the yield behaviour of the RVE and how it relates to the underlying yield property of the amorphous polymer.

### 3.2. Experimental

#### 3.2.1. Stress–strain Results

**Figure 3:**
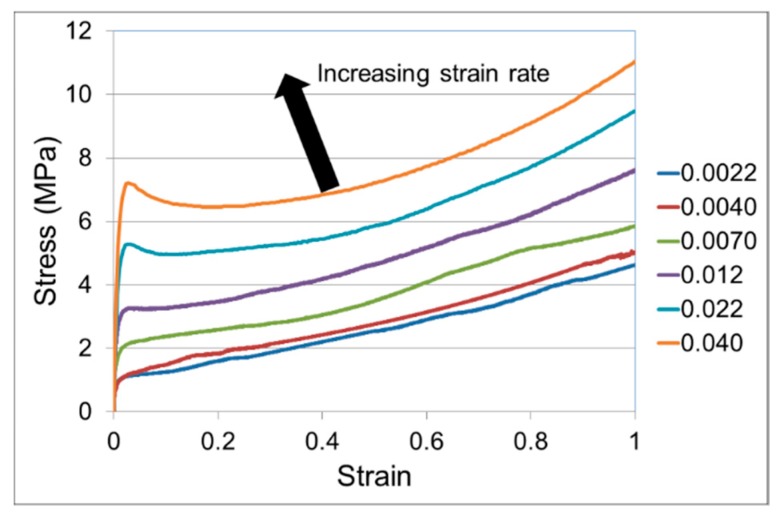
Stress–strain curves for the range of strain rates shown in s^−1^ in the caption.

A full set of stress–strain curves is shown in Figure 3. Each curve is an average of three results. The degree of reproducibility can be gauged from the yield stresses, for which the coefficient of variation is 7% or less, and the stresses at the end of loading, for which the coefficient of variation is 4% or less. There is recognisable yielding and strain hardening at all rates, and a marked strain rate dependence of stress, the latter also noted by Söntjens et al. [39] for a similar material. Zhou et al. [40] produced stress–strain curves of PLA over a range of temperatures at a strain rate of 0.0013 s^−1^. At 60 °C they report stresses higher than those at the lowest rate in Figure 3, consistent with their PLA material being of higher crystallinity. The initial linear response was detectable and measurable up to very low strain (0.1–0.2%). Since the strain was calculated from the Instron crosshead movement, its accuracy was considered to be insufficient to give reliable Young’s modulus values. Therefore, the tests were supplemented with three-point bend tests as described above, for which larger crosshead motions are required to achieve the strains within the linear range. From these measurements the modulus was found to be 0.53 GPa.

#### 3.2.2. Stress Relaxation

**Figure 4:**
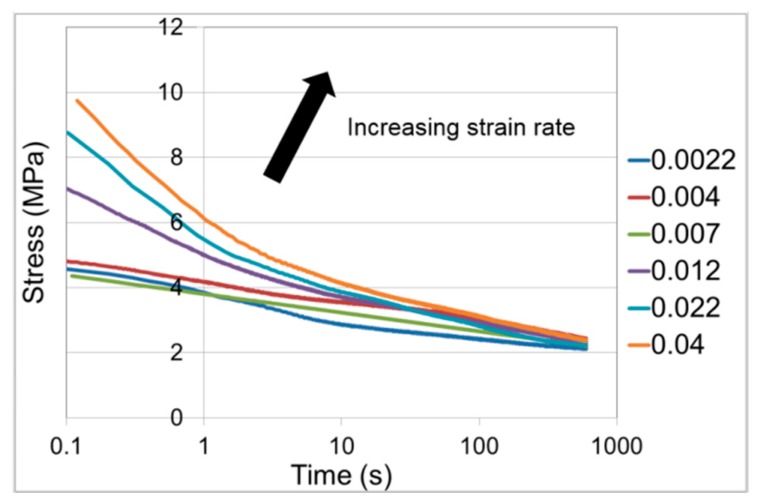
Stress relaxation for a range of initial strain rates shown in s^−1^ in the caption.

Initial loading was as described in Section 3.2.1 above, with the strain held constant for 1000–1400 s. Stress relaxation curves for the range of strain rates are shown in Figure 4. Each curve is an average of three results. For each set of three results, the coefficient of variation is less than 4% at the start of stress relaxation and has increased to be less than 7% by the end of the experiments.

There is little data on stress relaxation of PLA in the literature. Guedes et al. [41] report stress relaxation on a PLA-PCL (polycaprolactone) blend at room temperature and at strains up to 0.16, and were able to model the results using linear viscoelasticity. Duscunceli et al. [42] have studied stress relaxation of PLA at small strains and up to 50 °C. For conditions closer to those of this study, Sweeney et al. [43] report stress relaxation for a PLA-CaCO_3_ composite at a strain of 3.0 in the temperature range 60–70 °C, with initial loading at an average strain rate of 0.036 s^−1^. For these results at 60 °C, the stress at 1000 s has dropped from its initial value by 63%; this compares with the loading rate of 0.04 s^−1^ in Figure 4 in which the stress drops by 72% at 1000 s. On this basis, the stress relaxation behaviours are roughly equivalent.

#### 3.2.3. Strain Recovery under Load

**Figure 5:**
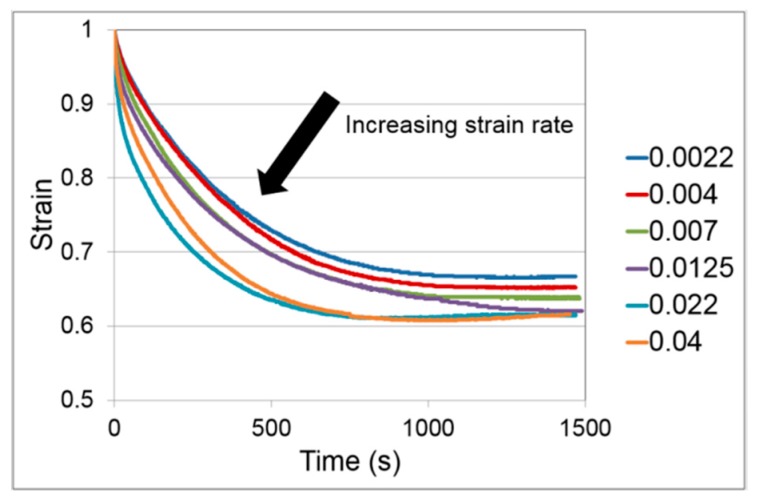
Strain recovery under load for a range of strain initial rates shown in s^−1^ in the captions, recovering at a load corresponding to an engineering stress of 0.89 MPa.

Initial loading was as described in Section 2 above, with a level of fixed load applied using a 5.9 N weight, corresponding to an engineering stress of 0.89 MPa. Images were captured at 1 s time intervals. Results are shown in Figure 5. Each curve is an average of three tests for which the coefficient of variation of the strain at 1400 s is 6% or less. Recovery levels are similar to those reported for a PLA-PBS blend when stretched to strains of 1.0, 2.0 and 2.5 [44].

### 3.3. Numerical Analysis of Results

#### 3.3.1. Effects of Inclusions

For this method of modelling, set out in 3.1.2 above, results are not affected by the absolute size of inclusions. However, their aspect ratios can have significant effects. We acknowledge that in practice crystal inclusions will possess a range of aspect ratios, whereas in the current modelling all the inclusions are identical. We have varied the aspect ratio in order to explore its significance on the overall stress–strain behaviour of the RVEs. A typical lamella crystal has a thickness d equal to the chain length along the c direction (see Figure 2), and along the other directions normal to c, (i.e., a and b), there are many chain folds, corresponding to lengths greater than d. We have assumed that the dimensions normal to c are both equal to w. The aspect ratio is defined as *d*/*w* and we have adopted three values, *d*/*w* = 0.34, 0.17 and 0.086.

Orthotropic elastic properties are assigned to each inclusion based on the X-ray derived moduli of Lee et al. [36] for PLLA and PLDA crystals. Along the chain axis, they derived the value *E*_c_ = 14 GPa, which we assign to the c direction of the inclusion. In the transverse directions a and b, they measured 3.2 and 2.8 GPa. Since these values are similar we have assumed the inclusions to be transversely isotropic, with transverse modulus *E*_t_ in the a–b plane given by *E*_t_ = 3 GPa. Lee et al. found no significant change in modulus in the temperature range 13–300 K, and with a melt temperature of 165 °C for our material, the room temperature moduli of Lee et al. are appropriate for our 60 °C tests. 

For the transversely isotropic inclusions the compliance matrix [45] is given in local a–b–c axes (see Figure 2) as

(8) $$S=\left(\begin{array}{ccc} s_{\mathrm{aa}} & s_{\mathrm{ab}} & s_{\mathrm{ac}}\\ s_{\mathrm{ab}} & s_{\mathrm{aa}} & s_{\mathrm{ac}}\\ s_{\mathrm{ac}} & s_{\mathrm{ac}} & s_{\mathrm{cc}} \end{array}\right)$$

for normal stresses and strains plus the shear compliance terms $s_{\mathrm{abab}},s_{\mathrm{bcbc}}$ which relate the shear stresses and strains:

(9) $$\begin{array}{l} \varepsilon_{\mathrm{ab}}=s_{\mathrm{abab}}\sigma_{\mathrm{ab}}\\ \varepsilon_{\mathrm{bc}}=s_{\mathrm{bcbc}}\sigma_{\mathrm{bc}}\\ \varepsilon_{\mathrm{ac}}=s_{\mathrm{bcbc}}\sigma_{\mathrm{ac}} \end{array}$$

Since ab is an isotropic plane, we have the relation [45]

(10) $$s_{\mathrm{abab}}=2\left(s_{\mathrm{aa}}-s_{\mathrm{ab}}\right)$$

The diagonal terms of Equation (8) are defined by

(11) $$\begin{array}{l} s_{\mathrm{aa}}=\frac{1}{E_{t}}\\ s_{\mathrm{cc}}=\frac{1}{E_{c}} \end{array}$$

and the off-diagonal terms depend on the Poisson’s ratios $\nu_{\mathrm{ba}},\nu_{\mathrm{ac}}$:

(12) $$\begin{array}{l} s_{\mathrm{ab}}=-\nu_{\mathrm{ba}}s_{\mathrm{aa}}=-\frac{\nu_{\mathrm{ba}}}{E_{t}}\\ s_{\mathrm{ac}}=-\nu_{\mathrm{ac}}s_{\mathrm{cc}}=-\frac{\nu_{\mathrm{ac}}}{E_{c}} \end{array}$$

We have generated values for $s_{\mathrm{ab}}$ and $s_{\mathrm{ac}}$ using Equations (12) on the assumption that $\nu_{\mathrm{ba}}=\nu_{\mathrm{ac}}=0.4$. This then yields a value for $s_{\mathrm{abab}}$ using Equation (10). No information is available for $s_{\mathrm{bcbc}}$. Two values for it are used of the same order of magnitude as $s_{\mathrm{abab}}$, but they differ from each other by 50% to investigate the effect of this variation on the RVE stress response.

The components of **S** generated in this way are

(13) $$S=\left(\begin{array}{ccc} 0.333 & -0.133 & -0.029\\ -0.133 & 0.333 & -0.029\\ -0.029 & -0.029 & 0.071 \end{array}\right){\left(\mathrm{GPa}\right)}^{-1}$$

for the normal stresses and strains and

(14) $$\begin{array}{l} s_{\mathrm{abab}}=0.933\;{\left(\mathrm{GPa}\right)}^{-1}\\ s_{\mathrm{bcbc}}=1.0,\;0.667\;{\left(\mathrm{GPa}\right)}^{-1} \end{array}$$

for the shear components.

For the ABAQUS analysis, stiffness components are required. These were obtained by inverting **S** and taking reciprocals of $s_{\mathrm{abab}}$ and $s_{\mathrm{bcbc}}$.

**Figure 6:**
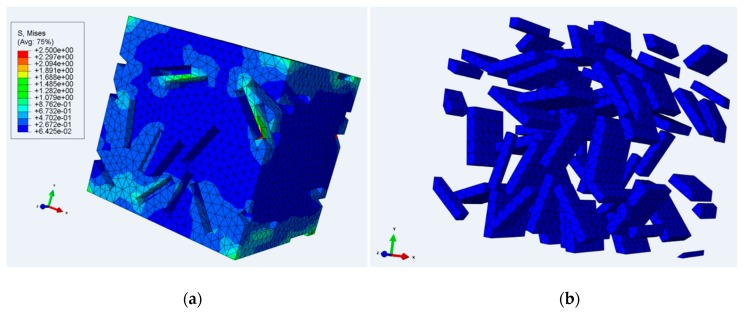
RVE realisation containing 43 inclusions with *d*/*w* = 0.17 and volume fraction φ = 0.13. (**a**) Model view without inclusions (**b**) showing inclusions only.

A typical realisation is shown in Figure 6.

First, the method was used to estimate the modulus at small strains of the amorphous phase. Linear elastic analysis was used. Several realisations were implemented for each inclusion aspect ratio, evaluating a stiffness matrix **C** for each run using Equations (5). The moduli E_1_ and E_2_ of the RVEs were taken as representative of the effect of randomly oriented inclusions, in contrast with E_3_ which is in the direction along which the inclusions were perfectly aligned. For each inclusion, aspect ratio the average value of E_1_ and E_2_ was derived, initially by varying the amorphous modulus *E*_a_ on a trial-and-error basis to attain an average modulus consistent with the observed value of 0.53 GPa. As a result, we found that the value *E*_a_ = 0.36 GPa gave a set of averaged values as given in Table 1.

From these results we conclude that *E*_a_ = 0.36 GPa is an adequate estimate of the amorphous phase modulus at the test temperature of 60 °C. The effect of the variation in $s_{\mathrm{bcbc}}$ on the overall modulus is within the range of error.

We have also used the RVE technique to explore the relationship between macroscopic stress–strain behaviour of the partially crystalline material and the yielding of the amorphous phase. As is apparent from Figure 3, deviation from linearity begins at low strains. For this analysis we have selected several RVEs and subjected them to uniaxial extension, having replaced the elastic material model of the amorphous phase with an elastic-plastic model governed by a von Mises criterion (Equation (7)) and allowing nonlinear deformations. We assume that the crystallite inclusions remain elastic as defined above. We assign values of uniaxial yield stress $\sigma_{Y}$ to the amorphous region of 1.0, 2.5, 5.0 and 10.0 MPa, covering the range of stresses observed in Figure 3 for the various strain rates.

**Figure 7:**
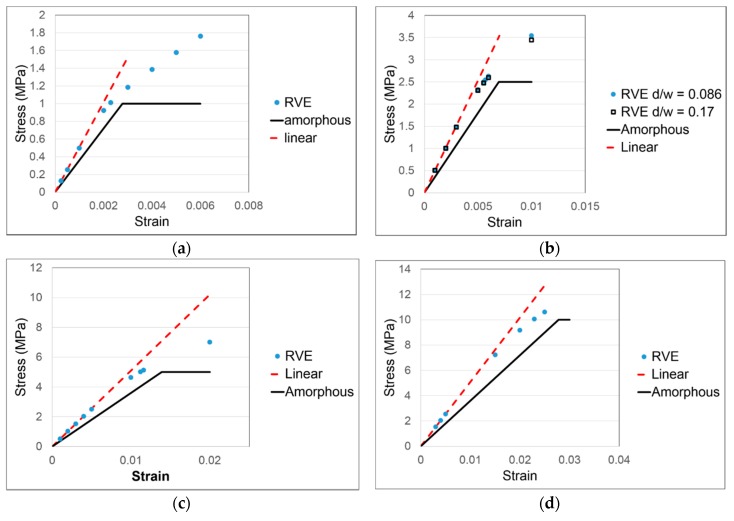
Comparison of RVE stress with linear extrapolation and stress-strain behaviour of the amorphous phase for yield stresses (**a**) 1.0; (**b**) 2.5; (**c**) 5.0; and (**d**) 10.0 MPa. Aspect ratios of inclusions are *d*/*w* = 0.086 except for (**b**) where aspect ratios of 0.086 and 0.17 have been compared.

In Figure 7, we show RVE results for the four values of yield stress. The macroscopic stress can be compared with the linear extrapolation of stress from low strains. For yield stresses 1.0, 2.5 and 5 MPa, there is a consistent relationship between the macroscopic stress, the yield stress and the linear extrapolation. For a macroscopic uniaxial stress $\sigma_{RVE}\left(\varepsilon \right)$ and linear extrapolation $\sigma_{lin}\left(\varepsilon \right)=E\varepsilon$, we can calculate a proportional difference *p* between the two for a given strain:

(15) $$p=\frac{E_{\varepsilon }-\sigma_{RVE}(\varepsilon )}{\sigma_{RVE}(\varepsilon )}$$

**Figure 8:**
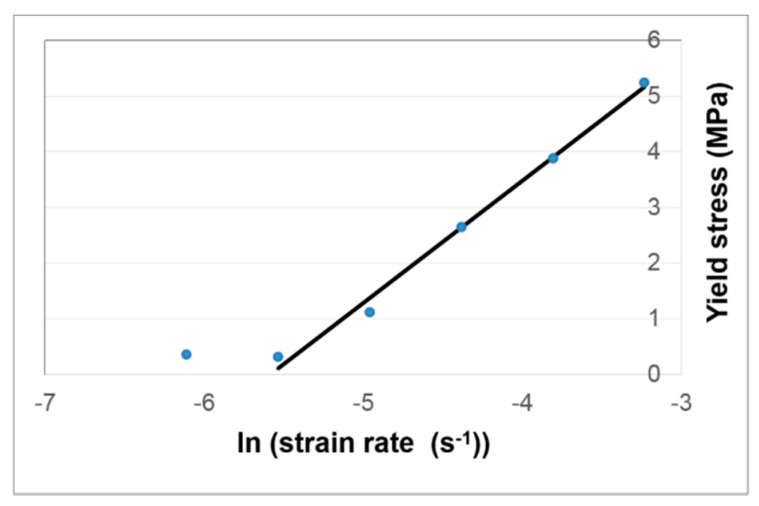
$\sigma_{Y}$ derived from the offset condition *p* = 0.138 as a function of strain rate.

At the particular strain when $\sigma_{RVE}\left(\varepsilon \right)=\sigma_{Y}$ values of *p* are given in Table 2. In the range 1–5 MPa yield stress, *p* is essentially constant. This phenomenon provides a means of detecting yield in the amorphous phase from an experimental stress–strain curve: when the offset of the stress from the linear extrapolation is at this constant value of *p*, the experimental stress is equal to the yield stress. In this region of stress, *p* is on average 0.138. The deviations from linearity seen in Figure 3 occur within the range of stress 1–5 MPa, so it is appropriate to use this value of *p* to calculate $\sigma_{Y}$ for the six strain rates plotted there. The $\sigma_{Y}$ values thus derived are plotted against logarithm of strain rate in Figure 8. Except for the lowest two strain rates, for which the stress–strain curves are essentially indistinguishable, the yield stresses show a linear dependence on the logarithm of rate. This in contrast to the corresponding plot for the maximum experimental stresses, which shows a distinct upward curvature.

#### 3.3.2. Modelling the Time-Dependent Yield of the Amorphous Phase

The linearity of response shown in Figure 8 suggests that yielding of the amorphous region is governed by an Eyring process [45,46]. In its simplest form, for uniaxial conditions, this process takes the form

(16) $${\dot{\varepsilon }}_{p}=\alpha \sinh\left(\frac{v}{kT}\sigma \right)$$

where ${\dot{\varepsilon }}_{p}$ is the plastic strain rate, α a temperature dependent factor, *v* the activation volume, *k* the Boltzmann’s constant and *T* the absolute temperature. For convenience we write $V=\frac{v}{kT}$, where V is the operational activation volume in units of reciprocal stress so that Equation (16) becomes

(17) $${\dot{\varepsilon }}_{p}=\alpha \sinh\left(V\sigma \right)$$

To model yield, it is assumed that the Eyring process acts in series with an elastic element to form a Maxwell-like model. Then, as total strain on this model is increased, stress increases until the plastic strain rate, rising as a nonlinear function of stress, matches the applied strain rate and the elastic element ceases to stretch. Then stress remains constant at a value dependent on the applied strain rate. We may use Equation (17) to model yield by equating the applied strain rate with the plastic strain rate. Also, the sinh function can be approximated with an exponential function on the assumption that the argument of the sinh is large enough. Then Equation (17) becomes

(18) $${\dot{\varepsilon }}_{p}=\frac{1}{2}\alpha \exp\left(V\sigma_{Y}\right)$$

and can be re-arranged as

(19) $$\sigma_{Y}=\frac{1}{V}\ln\left(\frac{2\dot{\varepsilon }}{\alpha }\right)$$

so that the linear slope in Figure 8 can be interpreted. The slope in Figure 8 gives a value *V* = 0.454 MPa^−1^, corresponding to an activation volume *v* = 2.1 nm^3^. This can be compared with the value 0.72 nm^3^ obtained by Söntjens et al. [39] for combined results of PLLA, PDLLA and PLDLLA.

Additionally, the value of α can be obtained from the intercept of the fitted line in Figure 8 to give the value α = 0.00752 s^−1^. This Eyring process is now fully defined.

The analysis so far has only covered the time-dependent behaviour at small strain. The stress relaxation results provides data for large strain, and can be analysed in terms of the Eyring process. Guiu and Pratt [47], again using the model of an Eyring process in series with a spring, showed that the approximation Equation (18) leads to a form of stress relaxation curve for stress at time t after the state of constant strain has been reached at time t = 0:

(20) $$\sigma \left(t\right)-\sigma \left(0\right)=\frac{1}{V}\ln\left(1+\frac{t}{c}\right)$$

Here, c is a constant that involves the stiffness of the elastic element, which is assumed to be linear elastic. For large strain Sweeney et al. [43] have generalised the model so that the elastic element operates at large strain and is in the form of a Gaussian model. The Maxwell model is assumed to be stretched at a constant extension ratio λ which is split multiplicatively into elastic and plastic components $\lambda_{e}$ and $\lambda_{p}$ respectively, so that

(21) $$\lambda =\lambda_{e}\lambda_{p}$$

The plastic strain rate is identified as

(22) $${\dot{\varepsilon }}_{p}=\frac{{\dot{\lambda }}_{p}}{\lambda_{p}}$$

Then, c is given by

(23) $$c=\frac{1}{\alpha \mathrm{VG}\left(2\lambda_{e}^{2}+\lambda_{e}^{-1}\right)}\exp\left(-V\sigma \left(0\right)\right)$$

where G is the strength of the Gaussian process and the stress in uniaxial conditions is given by

(24) $$\sigma =G\left(\lambda_{e}^{2}-\lambda_{e}^{-1}\right)$$

**Figure 9:**
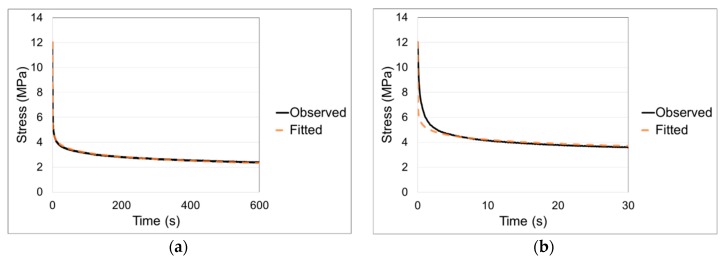
Stress relaxation for loading at 0.04 s^−1^ fitted to Equation (20). (**a**) Complete curve (**b**) Initial 30 s.

There is an approximation involved in the derivation of Equation (23), in that the quantity $2\lambda_{e}^{2}+\lambda_{e}^{-1}$ is assumed to be a constant; this means that the analysis is valid for slowly varying stress, and makes the evaluation of α from the fitted value of c difficult to justify. We have generated optimised fits of Equation (20) to the experimental stress relaxation curves of Figure 4 to give values of V and c. The theory set out above applies to stress relaxation following instantaneous loading and so we base the analysis on the highest strain rate of 0.04 s^−1^. The fitted and experimental curves are shown in Figure 9. The fitted parameter values are V = 2.2 MPa^−1^ and c = 3.47 × 10^−7^ s^−1^.

The Maxwell model comprising Gaussian and Eyring processes is to be used for the more general case of loading and strain recovery. For this analysis we require the Eyring stress as a function of plastic strain rate, which can be derived from Equation (17) as

(25) $$\sigma =\frac{1}{V}\ln\left[\frac{{\dot{\varepsilon }}_{p}}{\alpha }+\sqrt{{\left(\frac{{\dot{\varepsilon }}_{p}}{\alpha }\right)}^{2}+1}\right]$$

This is in equilibrium with the Gaussian element, so we have

(26) $$\frac{1}{V}\ln\left[\frac{{\dot{\varepsilon }}_{p}}{\alpha }+\sqrt{{\left(\frac{{\dot{\varepsilon }}_{p}}{\alpha }\right)}^{2}+1}\right]-G\left(\lambda_{e}^{2}-\lambda_{e}^{-1}\right)=0$$

To solve this, we express it in terms of the plastic extension ratio $\lambda_{p}$ which is related to the plastic strain rate by Equation (22). Also we eliminate $\lambda_{e}$ by using Equation (21), obtaining

(27) $$\frac{1}{V}\ln\left[\frac{1}{\alpha }\frac{{\dot{\lambda }}_{p}}{\lambda_{p}}+\sqrt{{\left(\frac{1}{\alpha }\frac{{\dot{\lambda }}_{p}}{\lambda_{p}}\right)}^{2}+1}\right]-G\left({\left(\frac{\lambda }{\lambda_{p}}\right)}^{2}-{\left(\frac{\lambda }{\lambda_{p}}\right)}^{-1}\right)=0$$

where λ is the total extension ratio applied to the model. Equation (27) is solved numerically using a time-incremental approach. At each increment, the value of $\lambda_{p}$ is calculated and the stress is then given by

(28) $$\sigma =G\left({\left(\frac{\lambda }{\lambda_{p}}\right)}^{2}-{\left(\frac{\lambda }{\lambda_{p}}\right)}^{-1}\right)$$

**Figure 10:**
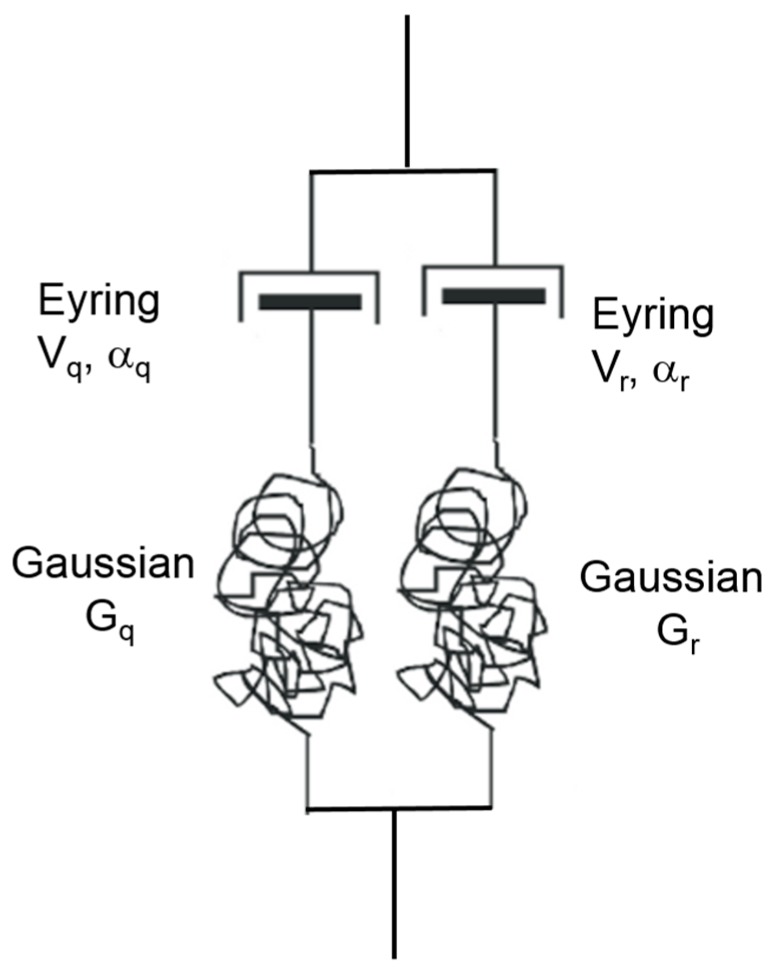
Two-process model.

To model the strain recovery experiments, two parallel Maxwell models are required (see Figure 10). The two arms are denoted by using subscripts q and r and each generates a stress, respectively $\sigma_{q}$ and $\sigma_{r}$ so that the total stress is given by

(29) $$\sigma_{tot}=\sigma_{q}+\sigma_{r}$$

Each arm is defined by Equations (27) and (28), with parameters $V_{q},V_{r},\alpha_{q},\alpha_{r},G_{q},G_{r}$ as appropriate. The total extension ratio λ is common to both arms. When λ is known, such as when the model is being strained at a constant rate (as in the initial loading of a strain recovery experiment), the solution is straightforward. When the total stress $\sigma_{tot}$ is known (as in the recovery phase of the experiment), Equations (27) to (29) are solved iteratively to produce the required stress.

The *q* arm of the model is associated with the Eyring process and stiffness identified in the analysis of loading behaviour. The operational activation volume $V_{q}$ was as obtained from the slope of the plot in Figure 8. The fitted value of $\alpha_{q}$ from the intercept in Figure 8 was found to give an unrealistically fast strain recovery and has been lowered from 0.00745 to 0.004 s^−1^. The Gaussian parameter $G_{q}$ is taken from the initial modulus for the amorphous phase of $E_{a}$ = 0.36 GPa. The small–strain behaviour of the Gaussian mechanism ensures that $G_{q}=E_{a}/3$. The *r* arm has a much lower stiffness and is associated with strain recovery; after the initial strain application, it remains in tension and causes recovery. Its stiffness is estimated from the stress and strain at the end of the stress relaxation experiments and assuming that they are related by a Gaussian model of strength $G_{r}$. The operational activation volume $V_{r}$ is taken from the stress relaxation fit of Figure 9. Due to the approximation involved in Equation (23) mentioned above, $\alpha_{r}$ cannot be evaluated from the fitted value of c and is varied by trial and error to produce realistic predictions.

**Figure 11:**
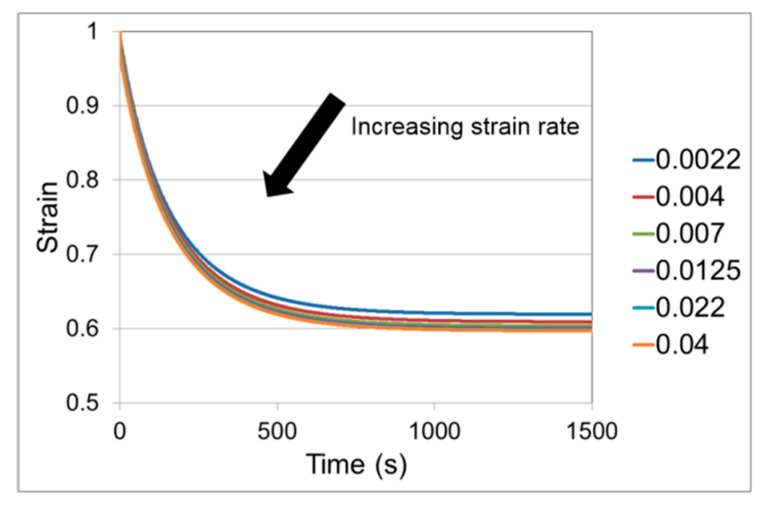
Strain recovery predictions of model in Figure 10 using parameters in Table 3.

The parameters used are summarised in Table 3. The predictions of recovery are given in Figure 11.

## 4. Discussion

**Figure 12:**
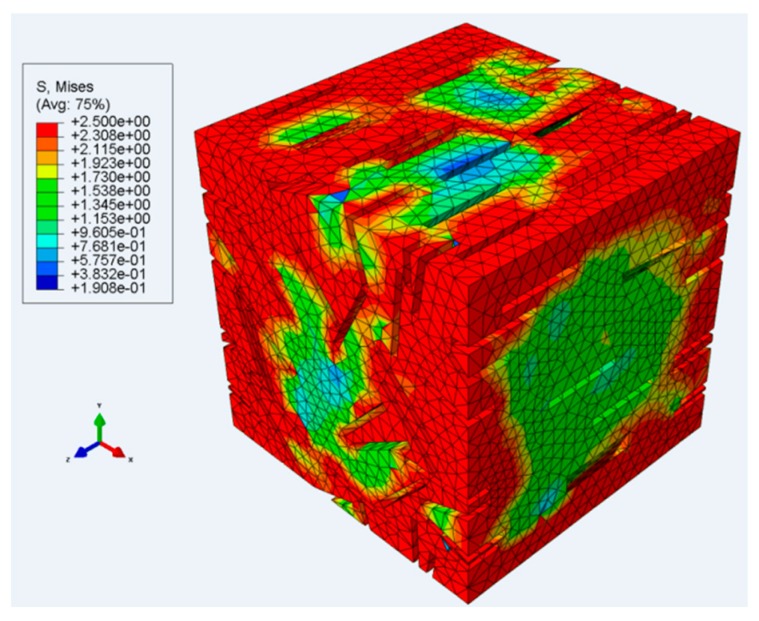
Finite element model of material with 89 inclusions at aspect ratio *d*/*w* = 0.086. Material yield stress is 2.5 MPa. Stress levels in MPa.

Elastic finite element modelling of the partially crystalline material shows that the crystal phase has a significant effect on its stiffness and allows us to estimate the Young’s modulus of the amorphous phase. The analysis has been extended to elastic–plastic behaviour of the amorphous phase, and a method has been developed to identify its yield stress from stress–strain measurements of the material. This method takes the form of an offset criterion, with the yield stress identified by the difference between the applied stress and the stress extrapolated from the linear elastic region. The overall stress in the model material continues to increase after the amorphous yield stress has been exceeded. In Figure 12, we show the inhomogeneous stress field in the amorphous material for the case in which the stress applied to the model is equal to the amorphous yield stress. In some regions the material has yielded, whereas elsewhere the stress is lower due to shielding of the crystal phase. The method has been used to define the strain–rate dependence of the amorphous yield stress, which is found to be consistent with an Eyring process.

We used the above analysis of yield and some stress relaxation data to help define the properties of a two-Eyring process model. This is clearly a simplification in comparison with the real material, which would be expected to include a spectrum of processes equivalent to many different Eyring parameters. The two-arm model comprises the minimum number of arms required to model recovery, when one arm is in tension and is the mechanism for recovery, while the other one resists the recovery and is in compression. The model parameters have in two cases been adjusted to give realistic recovery behaviour. Comparison of Figure 5 and Figure 11 shows that the level of recovery is approximately as observed, and, in common with the observations, increases as the strain rate of the initial stretch is increased. The effect of the initial strain rate is in practice greater than that predicted. The origin of the effect of strain rate is that, during loading, the stiff (q) arm is extended and the time available for the Eyring component to extend is less for higher strain rates. It therefore extends less and a greater proportion of the strain in the arm q is in the elastic element; there is therefore more potential for the model to recover. The other arm r does not contribute to the level of recovery as it resists recovery, being in compression. The dependence of the strain recovery on the initial strain rate could be increased by lowering the stiffness of the elastic part of the q arm, but this quantity is fixed by the observed material stiffness during loading. Introducing a third arm with stiffness lower than the q arm could remedy this problem; however, the extra parameters that would need to be specified would not be directly measurable and the model would have to be fitted by trial and error. Thus, it appears that this deficiency is a consequence of the model’s simplicity.

It should be noted that we have in effect made a material model of the amorphous phase only. The finite element modelling has the effect of isolating the yield behaviour of the amorphous phase to define the q arm, and the r arm is driven by entropic stress that is only a feature of the amorphous phase. This should be an adequate approach for modelling strain recovery behaviour, while more development would be needed to model levels of overall stress.

## Figures and Tables

**Table 1:** Average values of *E*_1_ and *E*_2_ calculated from RVEs. Errors are standard deviations.

Aspect Ratio *d*/*w*	N target Number of Inclusions	Number of Realisations	$s_{\mathrm{bcbc}}\;{\left(\mathrm{GPa}\right)}^{-1}$	Mean Modulus (GPa)
0.086	87	11	0.667	0.534 ± 0.017
0.086	87	12	1.0	0.523 ± 0.022
0.17	43	21	0.667	0.512 ± 0.035
0.34	22	21	0.667	0.507 ± 0.029

**Table 2:** Values of offset factor *p* as a function of yield stress.

$\sigma_{Y}\;\left(\mathrm{MPa}\right)$	*p*
1.0	0.140
2.5	0.134
5.0	0.139
10.0	0.156

**Table 3:** Values of parameters for the model of Figure 10.

Arm	V (MPa)^−1^	α (s^−1^)	G (MPa)
q	0.45	0.004	120
r	2.2	1.0 × 10^−6^	0.74

## References

[1] Avérous L. (2004). Biodegradable Multiphase Systems Based on Plasticized Starch: A Review. J. Macromol. Sci. Polym. Rev..

[2] Castro-Aguirre E., Iñiguez-Franco F., Samsudin H., Fang X., Auras R. (2016). Poly(lactic acid)—Mass production, processing, industrial applications, and end of life. Adv. Drug Delivery Rev..

[3] Athanasiou K.A., Niederauer G.G., Agrawal C.M. (1996). Sterilization, toxicity, biocompatibility and clinical applications of polylactic acid/polyglycolic acid copolymers. Biomaterials.

[4] Brkaric M., Baker K.C., Israel R., Harding T., Montgomery D.M., Herkowit H.N. (2007). Early failure of bioabsorbable anterial cerivcal fusion plates. J. Spinal Disord. Tech..

[5] Smit T.H., Engels T.A.P., Wuisman P.I.J.M., Govaert L.E. (2008). Time-dependent mechanical strength of 70/30 poly(l,dl-lactide): Shedding light on the premature failure of degradable spinal cages. Spine.

[6] Rebelo R., Fernandes M., Fangueiro R. (2017). Biopolymers in medical implants: A brief review. Procedia Eng..

[7] Xu J., Song J. (2015). Polylactic acid (PLA)-based shape-memory materials for biomedical applications. Shape Memory Polymers for Biomedical Applications.

[8] Farah S., Anderson D.G., Langer R. (2016). Physical and mechanical properties of PLA, and their functions in widespread applications—A comprehensive review. Adv. Drug Delivery Rev..

[9] Garlotta D.A. (2001). Literature review of Poly(Lactic Acid). J. Polym. Environ..

[10] Bergström J.S. (2016). Hayman D An Overview of Mechanical Properties and Material Modeling of Polylactide (PLA) for Medical Applications. Ann. Biomed. Eng..

[11] Al-Itry R., Lamnawar K., Maazouz A., Billon N., Combeaud C. (2015). Effect of the simultaneous biaxial stretching on the structural and mechanical properties of PLA, PBAT and their blends at rubbery state. Eur. Polym. J..

[12] Blair R.W., Dunne N.J., Lennon A.B., Menary G.H. (2018). Processing-property relationships of biaxially stretched poly(l-lactic acid) sheet for application in coronary stents. J. Mech. Behav. Biomed. Mater..

[13] Wang X., Pan Y., Liu X., Liu H., Li N., Liu C., Schubert D.W., Shen C. (2019). Facile Fabrication of Superhydrophobic and Eco-Friendly Poly(lactic acid) Foam for Oil−Water Separation via Skin Peeling. ACS Appl. Mater. Interfaces.

[14] Chacón J.M., Caminero M.A., García-Plaza M., Núnez P.J. (2017). Additive manufacturing of PLA structures using fused deposition modelling: Effect of process parameters on mechanical properties and their optimal selection. Mater. Des..

[15] Coates P.D., Ward I.M. (1979). Drawing of Polymers through a Conical Die. Polymer.

[16] Coates P.D., Ward I.M. (1981). Die Drawing—Solid-Phase Drawing of Polymers through a Converging Die. Polym. Eng. Sci..

[17] Ward I.M., Taraiya A.K., Coates P.D. (2000). Solid state extrusion and die drawing. Solid Phase Processing of Polymers.

[18] Li J., Li Z., Ye L., Zhao X., Coates P.D., Caton-Rose F. (2017). Structure evolution and orientation mechanism of long-chain-branched poly(lactic acid) in the process of solid die drawing. Eur. Polym. J..

[19] Li J., Li Z., Ye L., Zhao X., Coates P.D., Caton-Rose F. (2017). Structure and biocompatibility improvement mechanism of highly oriented poly(lactic acid) produced by solid die drawing. Eur. Polym. J..

[20] Vasile C., Stoleru E., Darie-Niţa R.N., Dumitriu R.P., Pamfil D., Tarţau R. (2019). Biocompatible Materials Based on Plasticized Poly(lactic acid), Chitosan and Rosemary Ethanolic Extract I. Effect of Chitosan on the Properties of Plasticized Poly(lactic acid) Materials. Polymers.

[21] Li Q., Liu C., Wena J., Wua Y., Shana Y., Liao J. (2017). The design, mechanism and biomedical application of self-healing hydrogels. Chin. Chem. Lett..

[22] Ye A., Wang S., Zhao Q., Wang Y., Liu C., Shen C. (2019). Poly(ethylene oxide)-promoted dispersion of graphene nanoplatelets and its effect on the properties of poly(lactic acid)/poly(butylene adipate-co-terephthalate)-based nanocomposites. Mater. Lett..

[23] Peponi L., Navarro-Baena I., Kenny J.M., Aguilar M.R., Román J.S. (2014). Shape memory polymers: Properties, synthesis and applications. Smart Polymers and Their Applications.

[24] Fillon T.M., Xu J., Prasad M.L., Song J. (2011). In Vivo Tissue Responses to Thermal-responsive Shape Memory Polymer Nanocomposites. Biomaterials.

[25] Walzac J., Sobota M., Chrzanowski M., Krucinska I. (2018). Application of the melt-blown technique in the production of shape-memory nonwoven fabrics from a blend of poly(l-lactide) and atactic poly[(R,S)-3-hydroxy butyrate]. Text. Res. J..

[26] Zhang H., Wang S., Zhang S., Ma R., Wang Y., Cao W., Liu C., Shen C. (2017). Crystallization behavior of poly(lactic acid) with a self-assembly aryl amide nucleating agent probed by real-time infrared spectroscopy and X-ray diffraction. Polym. Test..

[27] Peltoniemi H., Kontio R., Lindqvist C., Suuronen R. (2002). The use of bioabsorbable osteofixation devices in craniomaxillofacial surgery. Oral Surg. Oral Med. Oral Pathol..

[28] Halpin J.C., Kardos J.L. (1972). Moduli of crystalline polymers employing composite theory. J. Appl. Phys..

[29] Halpin J.C., Kardos J.L. (1976). The Halpin-Tsai equations: A review. Polym. Eng. Sci..

[30] Kong Y., Hay J.N. (2002). The measurement of crystallinity of polymers with DSC. Polymer.

[31] Schindelin J., Arganda-Carreras I., Frise E. (2012). Fiji: An open-source platform for biological-image analysis. Nat. Methods.

[32] Mori T., Tanaka K. (1973). Average stress in matrix and average elastic energy of materials with misfitting inclusions. Acta Metall..

[33] Tucker C.L., Liang E. (1999). Stiffness predictions for unidirectional short-fiber composites: Review and evaluation. Compos. Sci. Technol..

[34] Spencer M.W., Paul D.R. (2011). Modeling the mechanical and thermal expansion behavior of TPO-based nanocomposites. Polymer.

[35] Mura T. (1991). Micromechanics of Defects in Solids.

[36] Lee S., Kimoto M., Tanaka M., Tsuji H., Nishino T. (2018). Crystal modulus of poly(lactic acid)s, and their stereocomplex. Polymer.

[37] Tábi T., Sajó I.E., Szabó F., Luyt A.S., Kovács J.G. (2010). Crystalline structure of annealed polylactic acid and its relation to processing. Express Polym. Lett..

[38] Press W.H., Flannery B.P., Teukolsky S.A., Vetterling W.T. (1986). Numerical Recipes, the Art of Scientific Computing.

[39] Söntjens S.H.M., Engels T.A.P., Smit T.H., Govaert L.E. (2012). Time-dependent failure of amorphous poly-d,l-lactide: Influence of molecular weight. J. Mech. Behav. Biomed. Mater..

[40] Zhou C., Guo H., Li J., Huang S., Li H., Meng Y., Yu D., de Claville Christiansen J., Jiang S. (2016). Temperature dependence of poly(lactic acid) mechanical properties. RSC Adv..

[41] Guedes R.M., Singh A., Pinto V. (2017). Viscoelastic Modelling of Creep and Stress Relaxation Behaviour in PLA-PCL Fibres. Fibers Polym..

[42] Dusunceli N., Drozdov A.D., Theilgaard N. (2017). Influence of Temperature on Viscoelastic–Viscoplastic Behavior of Poly(lactic acid) Under Loading–Unloading. Polym. Eng. Sci..

[43] Sweeney J., Bonner M., Ward I.M. (2014). Modelling of loading, stress relaxation and stress recovery of a shape memory polymer. J. Mech. Behav. Biomed. Mater..

[44] Tcharkhtchi A., Abdallah-Elhirsti S., Ebrahimi K., Fitoussi J., Shirinbayan M., Farzaneh S. (2014). Some New Concepts of Shape Memory Effect of Polymers. Polymers.

[45] Ward I.M., Sweeney J. (2013). Mechanical Properties of Solid Polymers.

[46] Halsey G., White H.J., Eyring H. (1945). Mechanical properties of textiles, I. Text. Res. J..

[47] Guiu F., Pratt P.L. (1964). Stress relaxation and the plastic deformation of solids. Phys. Status Solidi..

